# A U-Pb zircon age constraint on the oldest-recorded air-breathing land animal

**DOI:** 10.1371/journal.pone.0179262

**Published:** 2017-06-28

**Authors:** Stephanie E. Suarez, Michael E. Brookfield, Elizabeth J. Catlos, Daniel F. Stöckli

**Affiliations:** 1 Department of Geological Sciences, Jackson School of Geosciences, University of Texas at Austin, Austin, Texas, United States of America; 2 Environmental, Earth, and Ocean Sciences, University of Massachusetts at Boston, Boston, Massachusetts, United States of America; Institute of Botany, CHINA

## Abstract

The oldest-known air-breathing land animal is the millipede *Pneumodesmus newmani*, found in the Cowie Harbour Fish Bed at Stonehaven, Scotland. Here we report the youngest, most concordant ^238^U-^206^Pb zircon age from ash below the fish bed of 413.7±4.4 Ma (±2σ), whereas the youngest age from a tuffaceous sandstone above the fish bed is statistically indistinguishable at 414.3±7.1 Ma. The Cowie Harbour Fish Bed thus appears to be lowermost Devonian (Lochkovian), contrary to the previously accepted mid-Silurian age based on palynomorphs from adjacent exposures. This has implications for the evolutionary timetable of land colonization, as the Cowie ages overlap late Lochkovian zircon ages reported elsewhere for andesite below the nearby (~50 mi) Rhynie Chert, which has more advanced terrestrial biota. The results postdate the possible late Silurian Ludford Lane locality in Shropshire, England. *Pneumodesmus newmani* is thus not the earliest air-breathing land animal, unless the Ludford Lane locality is younger than presently assigned.

## Introduction

**Fig 1:**
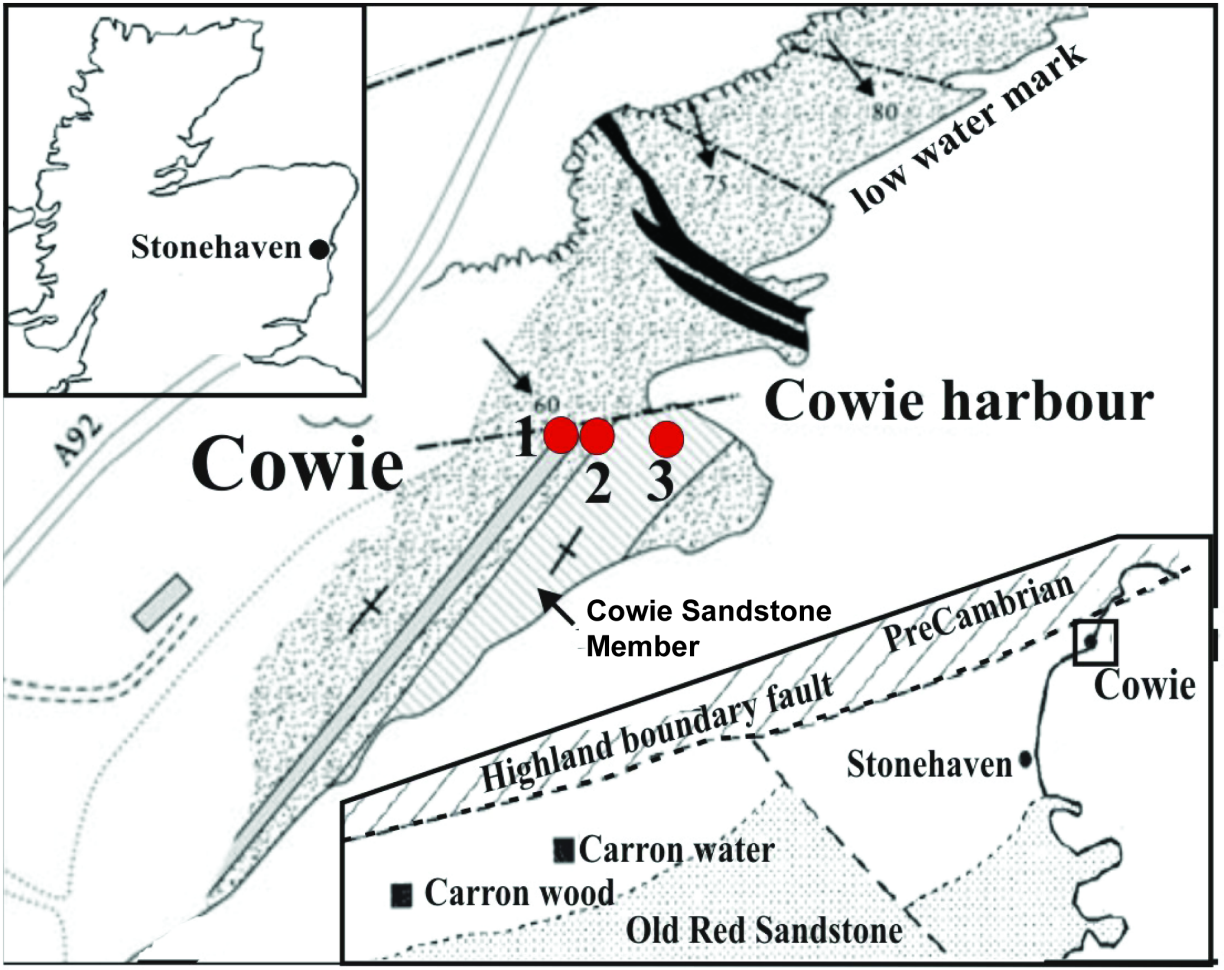
Location map of samples at Cowie Harbor, Scotland. The top left onset shows the location of Stonehaven. The bottom right highlights the relationship of inland exposures to Cowie and location of large map after [5]. Fig 1 is based on [5], which is included as S1 Fig.

Evaluating the rate and character of land colonization requires precise dating of early land biotas—but this is often difficult because of the problem of correlating and dating non-marine deposits. The supposed oldest air-breathing land animal found to date, the millipede *Pneumodesmus newmani*, is from the Cowie Harbour Fish Bed at Cowie, Aberdeenshire, Scotland [1, 2] (Fig 1). It is currently assigned a late Wenlock to early Ludlow age (mid-Silurian, ~427 Ma) based on plant spores from supposedly correlative, but tectonically isolated, and undescribed small exposures of sediment at Carron wood and Carron water inland (Fig 1) [1, 3, 4]. Fish and arthropod fossils from the Cowie Harbour Fish Bed itself suggest a Pridoli (latest Silurian) age of 423–419 Ma [5]. No spores are recorded from the Cowie Harbour Fish Bed, which is unexpected if correlative with the inland exposures due to its close proximity.

**Fig 2:**
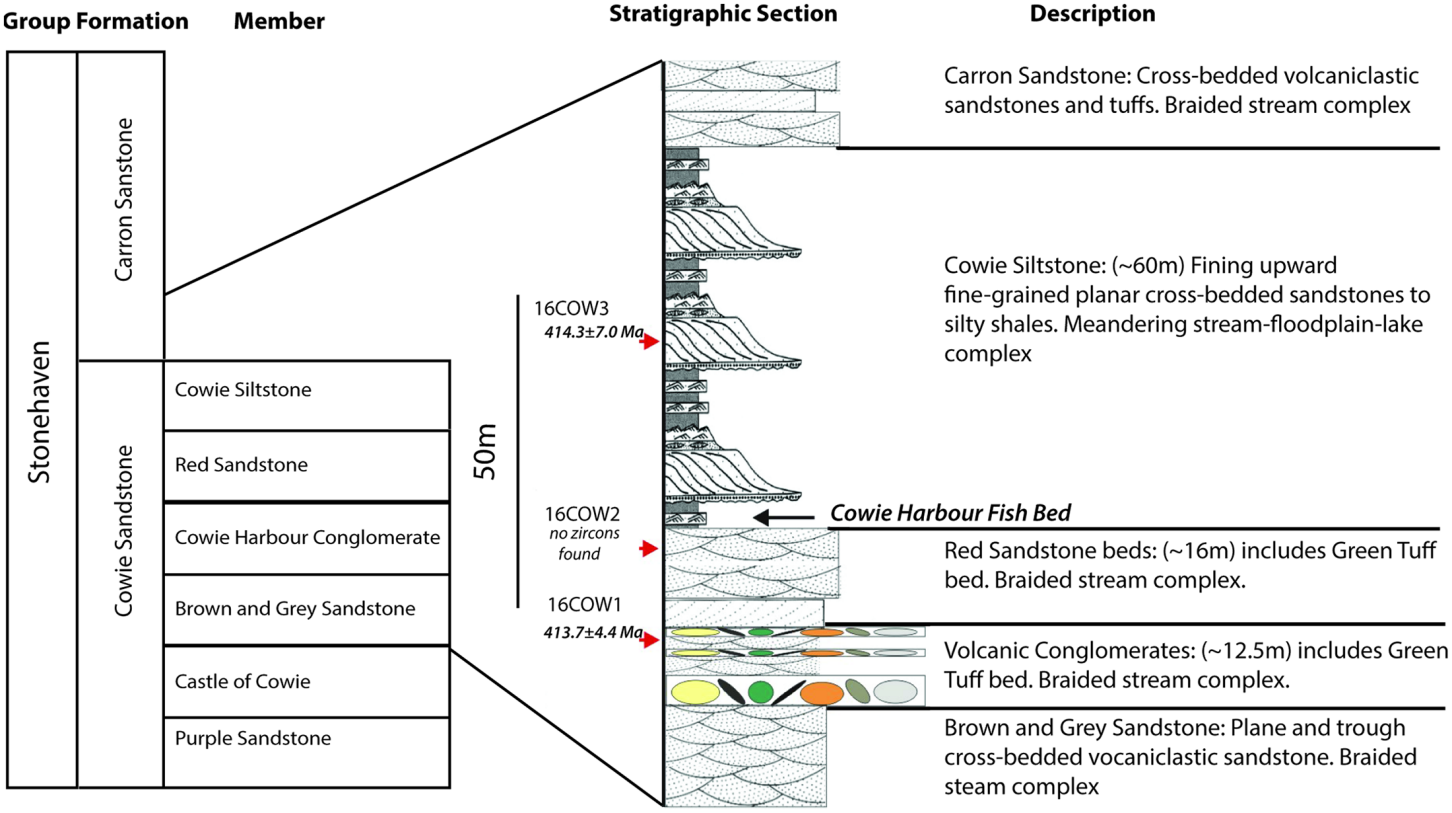
Stratigraphic section at Cowie and location of samples (16COW1, 16COW2, and 16COW3, indicated by 1, 2, and 3 respectively) based on information in [5] and [6]. Descriptions of the rock assemblages are included.

No radiometric dates exist from the succession, thus we applied U-Pb Laser Ablation-Inductively Coupled Plasma Mass Spectrometry (LA-ICP-MS) to zircon grains extracted from adjacent beds above and below the Cowie Harbour Fish Bed (Fig 2). Zircon ages from these units have important implications for the development and rate of evolution of early land communities. The results suggest rapid evolution of integrated invertebrate-plant land communities in the earliest Devonian.

## Setting and methods

The Cowie Harbour Fish Bed occurs in the tectonically isolated, but unmetamorphosed, non-marine succession of the Cowie Sandstone Formation, the older of two formations within the Stonehaven Group (Figs 1 and 2). The Cowie Sandstone Formation is composed of six members [6] and the youngest, the Cowie Harbour Siltstone, contains the Cowie Harbour Fish Bed at its base. The Stonehaven Group is characterized by alternating grey sandstones and mudstones interpreted as a river and floodplain complex [3, 5]. We collected three hand-sized samples for zircon U-Pb geochronology using LA-ICP-MS. Note that no specific permissions are required for sampling the rocks at the Cowie site. We followed the Scottish Outdoor Access Code published by Scottish Natural Heritage [7], and responsible collecting of material from the area is permitted. This is also stated specifically in [8]. The field studies did not involve endangered or protected species.

**Fig 3:**
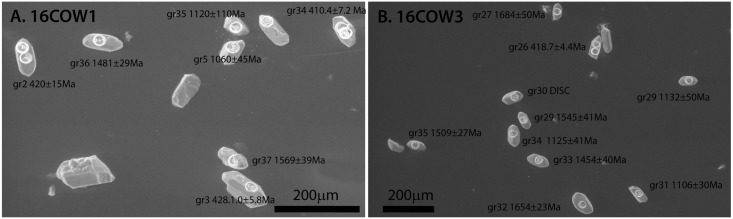
Secondary electron images of selected zircons from samples (A) 16COW1 and (B) 16COW3. Grain number and ages are indicated.

Sample 16COW1 is from a fine-grained green tuff at the top of the Cowie Harbour Conglomerate member, whereas sample 16COW2 is from a coarse bed within the Red Sandstone member directly below the fish bed. Sample 16COW3 is from a fine-grained tuffaceous sandstone located within the Cowie Harbour Siltstone member 20m above the fish bed. Using standard heavy-mineral separation methods, sample 16COW2 surprisingly contained no zircons, but the others supplied >100 grains that are typically 50–100μm in length (Fig 3). One explanation for the absence of zircon in 16COW2 is that small, fine sand-sized zircons remained in suspension in the higher energy environment indicated by the coarse grain-size of the red sandstone [9]. The zircon absence in 16COW2 and the abundant small grain sizes seen in 16COW1 and 16COW3, also suggest that only fine-grained zircons may have been available for transport, and thus Cowie may have been a long distance from the eruption source.

Forty zircon grains each from sample 16COW1 and 16COW3 were selected and dated using an Element2 High Resolution (HR)-ICP-MS with an Excimer (192 nm) laser ablation system instrumentation in the Geo-Thermochronometry lab at the University of Texas at Austin. Each zircon was mounted on double-sided tape, and was examined optically during the mounting process to select euhedral grains and eliminate analysis of cracked or metamict zircons (Fig 3). In this dating method, an ablated dry aerosol is introduced into the High Resolution ICP-MS using ultra-high purity He carrier gas for ^238^U, ^235^U, ^232^Th and ^206^Pb-^208^Pb isotopic measurements using ion-counting. Each analysis consisted of a 2-pulse cleaning ablation, a background measurement taken with the laser off, a 30 sec measurement with the laser firing, and a 30 sec cleaning cycle. The laser beam was 30μm diameter. Common Pb was corrected using the measured ^204^Pb (Hg-corrected) and assuming initial composition reported by [10]. Elemental and isotopic fractionation of Pb/U and Pb isotopes, respectively, is corrected by interspersed analysis of primary and secondary zircon standards with a known age (GJ1 and Plesovice) [11, 12]. The common unknown to standard measurement ratio was generally 3:1 or 4:1. Uncertainty resulting from calibration correction is 1–2% for both ^206^Pb/^207^Pb and ^206^Pb/^238^U. We attempted to date the youngest grains in each sample a second time, but the results were discordant. The youngest grains in Fig 3 have two laser ablation spots.

Data is summarized in Tables 1 and 2, with details provided in S1 Table. We report the ^238^U-^206^Pb age if the zircon was less than 850 Ma, and the ^207^Pb-^206^Pb age if the grain was older than 850 Ma. Ages were filtered for concordance, and ^238^U-^206^Pb ages that are >10% discordant are not reported (N = 9 out of 83 total). In addition to the age data, U (ppm) and U/Th for each analysis were obtained. Note that the whole zircon grains, as opposed to exposed zircon cross sections, were analyzed due to their small size. The time-resolved laser ablation technique allows us to evaluate data that is obtained as the laser drills continuously into the grain, thus identifying zones of different age and limiting the possibility of mixed ages. This process allows us to distinguish between zircon cores and rims, as different age populations as easily identified. All ages are reported with ±2σ uncertainty.

**Table 1:** LA-ICP-MS results from zircons dated in upper bed sample 16COW3.

Grain #(-Rim or core)^a^	U (ppm)	U/Th	Age (Ma)^b^	±2σ	% Discordance^c^
Devonian zircon ages					
4	190	0.9	414.3	7	0.65
9-Rim	241.7	1.27	415.0	12	0.97
26	275	0.82	418.7	4.4	1.48
Older zircon ages					
3	287	1.19	426.0	10	2.52
37	118.6	1.12	447.7	7.8	0.61
23	525	1.5	454.0	13	2.78
22	351	1	457.6	9.7	1.17
17	243	1.21	470.5	4.6	3.13
9-Core	172.1	1.85	651	18	7.26
11	83.3	1.13	940	76	0.53
19	123	1.62	1027	68	4.77
10	265	2.5	1041	32	1.06
18	298	1.99	1080	30	1.81
31	816	2.2	1106	30	1.36
7	377	1.8	1108	25	2.71
34	143.8	1.15	1125	41	3.64
28	580	7.71	1132	50	2.74
1	227.2	1.33	1156	47	2.94
2	200	2.31	1176	49	1.45
38	313	2.72	1298	35	22.34
8	116	0.67	1331	51	19.16
5	74.9	1	1345	51	1.49
14	320	2.43	1363	30	4.99
16	278	1.2	1385	24	0.07
33	153.9	2.09	1454	40	0.41
35	336	3.05	1509	27	1.59
20	369	2.29	1518	34	10.67
29	258	2.18	1545	41	1.94
24	399	2.64	1549	26	5.81
39	190.6	0.51	1605	23	2.49
13	161	1.11	1609	32	7.27
15	527	5.18	1620	33	14.01
32	204	1.29	1654	23	0.36
12	227.4	1.63	1657	21	1.27
27	85.1	2.33	1684	50	2.02
40	203.9	1.15	1688	27	4.32

^a.^ Number is the zircon grain that was dated, “-core” or “-rim” indicates that an age gradient is found through depth profiling.

^b.^ We report the ^238^U-^206^Pb age if the zircon is <850 Ma, and the ^207^Pb-^206^Pb age if the grain is >850 Ma.

^c.^ Reports the percent discordance, and is the ^206^Pb/^238^U discordance if the age is younger than 850 Ma and is the ^207^Pb/^206^Pb discordance age if grain is older than 850Ma

**Table 2:** LA-ICP-MS results from zircons dated in lower bed sample 16COW1.

Grain #	U (ppm)	U/Th	Age (Ma)^a^	±2σ	% Discordance
Devonian zircon ages					
34	570	2.96	410.4	7.2	3.37
6	596	2.8	413.7	4.4	0.72
1	628	2.54	413.8	6.3	5.11
12	713	2.5	414.0	13	1.9
14	934	2.65	414.0	12	3.72
27	411	2.67	415.0	9.2	2.58
19	468	1.36	418.8	4.6	0.88
Older zircon ages					
2	513	2.7	420.0	15	0.47
8	137	1.81	422.9	6.7	0.73
15	651	2.14	425.2	5.4	0.86
11	682	1.14	426.0	5.5	0.88
30	594	2.42	427.2	7.8	5.13
3	596	2.57	428.1	5.8	0.83
17	429.8	2.32	428.7	4.9	0.42
7	443	2.44	437.5	5.4	5.32
28	379	2.29	439.0	12	0.45
10	188.7	1.49	442.0	11	0
20	896	2.78	451.1	9.6	1.46
16	2340	3.18	460.1	7.7	1.65
33	1362	3.3	469.8	5	0.82
26	489	2.11	500.4	6.5	3.86
13	1874	61.6	580.6	7.5	9.8
41	20.49	0.23	728	18	7.96
29	77.1	1.26	875	85	0.57
23	111.2	1.14	1016	45	1.77
5	317	3.96	1060	45	7.64
18	186	1.4	1077	32	0.2
25	374.6	13.72	1093	38	6.22
32	282	2.43	1095	31	2.19
35	68.1	0.68	1120	110	17.32
21	461	1.03	1316	26	0.08
40	135	0.92	1463	46	5.26
36	634	8.4	1481	29	0.2
24	364	1.13	1499	45	10.21
42	284.9	3.04	1635	33	0.43
37	131.7	3.18	1569	39	19.95
38	272	1.17	1651	34	7.45
22	450	11.3	2684	32	5.66

^a.^ We report the ^238^U-^206^Pb age if the zircon is <850 Ma, and the ^207^Pb-^206^Pb age if the grain is >850 Ma.

^b.^ Reports the percent discordance, and is the ^206^Pb/^238^U discordance if the age is younger than 850 Ma and is the ^207^Pb/^206^Pb discordance age if grain is older than 850Ma.

## Results

Overall, 38 zircon ages are reported from sample 16COW1 and 36 ages from 16COW3. Ages from 16COW1 range from 2684±32 Ma (5.66% discordant) to 410.4±7.2 Ma (3.37% discordant), whereas those from 16COW3 range from 1688±27 (4.32% discordant) to 414.3±7.1 Ma (0.65% discordant). We found ten Devonian zircon ages (N = 7 from 16COW1 and N = 3 from 16COW3) and eleven that yield Silurian results (N = 10 from 16COW1 and N = 1 from 16COW3). The average Devonian ages from 16COW1 is 414.2±8.7 Ma (MSWD = 0.82), whereas the Devonian results from sample 16COW3 average 416.0±8.4 Ma (MSWD = 0.64). One Devonian age in sample 16COW3 (415.0±12.0 Ma) is found as a rim on a 651±18 Ma core. This is the only zircon that contains an older core and younger rim; the remainder of the grains did not show age gradients using our filters for discordance. The majority of the zircon grains analyzed are older than Silurian (55% of all results from 16COW1 and 89% of all results in 16COW3). We find no correlation between zircon age and U (ppm) or U/Th. The U/Th data is consistent with igneous zircons (e.g., [13]).

## Discussion

**Fig 4:**
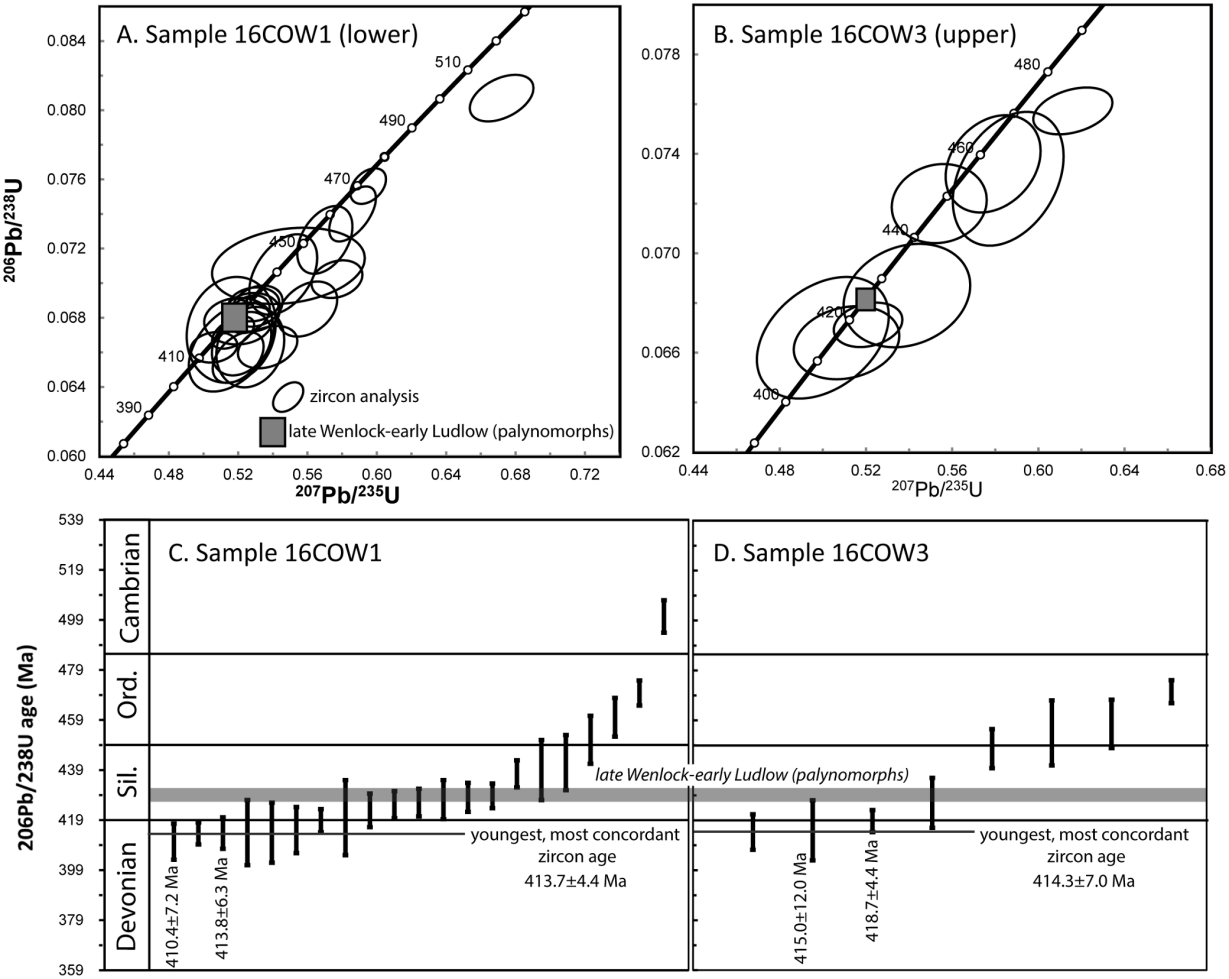
Concordia diagrams for zircon ages between 510 and 400 Ma for samples (A) 16COW1 and (B) 16COW3. (C) and (D) are plots of the ages in ascending order.

The weighted means of zircon ages from volcanic ashes, which are frequently used to define bed ages, are averages of the ages of zircons blown out by the explosive eruption which produced the ash, and must include some older material from the volcanic pile and country rock. Ultimately, they do not give the age of the eruption, but only an older age, depending on the amount of earlier material incorporated [14, 15]. They are meaningless in terms of the age of eruption of the ash that contains them [15]. Thus, we use the youngest, most concordant zircon ages as the maximum age of each bed, since a bed cannot be older than the youngest thing in it, although it can be younger (Fig 4; Tables 1 and 2). The youngest, most concordant U-Pb zircon age from the lowermost bed is 413.7±4.4 Ma (grain 6, Table 2), whereas the uppermost bed is 414.3±7.1 Ma (grain 4, Table 2). These are statistically indistinguishable, indicating rapid sedimentation, consistent with the paleo-environment.

The Cowie Harbour Fish Bed thus appears to be ~414 Ma in age, almost 13 million years younger than the currently accepted age. The younger zircon ages have the potential to be due to Pb loss from original mid-Silurian grains. However, zircons are often used to time major events in Earth’s history because they retain their daughter isotopes under a range of extreme conditions. If the ~414 Ma zircons were originally ~427 Ma, the zircons would have had to experience ~4% radiogenic Pb loss. Although this is a remote possibility, we selected clear, prismatic grains that lacked metamict features or textures suggestive of Pb loss. In addition, Fig 4C and 4D shows that a significant number of zircons yield the younger results, which requires they be considered when evaluating the age of *Pneumodesmus newmani*.

The uncertainty in the ages obtained using LA-ICP-MS is larger than ages that would be obtained using other approaches, such as isotope dilution thermal ionization mass spectrometry (ID-TIMS) analyses. The ID-TIMS approach applied to the youngest zircons would yield more precise ages since the two-sigma precision of individual ID-TIMS analyses is 0.1–0.3% compared with about 1–3% of individual LA-ICP-MS [16]. However, the only zircons available in the age range are small and aliquots of multiple grains would likely include the higher amounts of older material. We attempted a second LA-ICP-MS analysis of the youngest grains in the samples, but these yielded only discordant results. We anticipate that the TIMS analysis is almost certain to incorporate material from more than one zone and it is impossible to mechanically separate these in such small grains.

The Silurian-Devonian boundary is dated at 419.2±3.2 Ma [17]. Therefore, the Cowie Harbour Fish Bed and its air-breathing millipede are lowermost Devonian (Lochkovian) in age, contrary to the previously accepted Silurian age—on the current time scale. This has important implications for the evolutionary timetable of land colonization, as the Cowie age range statistically overlaps a U-Pb zircon age of 411.5±1.3 Ma (late Lochkovian, average of N = 4 grains, TIMS) for an andesite just below the Rhynie Chert with its more advanced and better preserved terrestrial biota [18] and is possibly younger than the supposed land centipedes and arachnid recorded from the Ludford Lane bone bed [19, 20], which is correlated with the basal Pridoli (423±2.3 Ma). However, the biostratigraphic inferred ages of such early freshwater and land biotas are based on correlation with marine sections whose radiometric dating requires revision.

The fish/arthropod fauna of the Stonehaven Group, within which the Cowie Fish Bed is located, differs dramatically from those at structurally shallower levels of the Arbuthnott Group in the Midland Valley (see [21] for cross-section). Fault systems and unconformities may exist throughout the section in Fig 2 that are currently not exposed, and the Stonehaven Group may be of a separate provenance and depositional succession and structurally juxtaposed into its current position [22]. This observation leaves the significance of the dates here unaffected, but should be considered in interpretations for the structural development of the region.

Although attention has been paid to ascertaining ages to successions in Fig 2 via other means, consensus on the age of the units has not been achieved (e.g., [23], see Discussion regarding dating exposures of the Ringerike Group (Old Red Sandstone) in Norway). Many recent calibrations of the geological time scale have relied heavily on statistical techniques on conjunction with best-fit line techniques for estimating numerical ages for chronostratigraphic boundaries. For example, the Silurian time scale is based on calibrating a constrained optimization (CONOP) composite graptolite zonation to selected radiometric ages (e.g., [24]). But this, while useful, is no substitute for precise radiometric dating of ashes in fossiliferous sections if available.

## Supporting information

S1 Table Description of the excel file: Detailed U-Pb LA-ICP-MS data. The first tab (Explanation) is a detailed key for the raw data presented under the second tab (Data).
(XLSX)

S1 Fig Geological sketch-map of the coast of north of Stonehaven from [5]. (TIF)
