# Supplementary figures and images for: A U-Pb zircon age constraint on the oldest-recorded air-breathing land animal
Source: PLoS One. 2017 Jun 28;12(6):e0179262. doi: 10.1371/journal.pone.0179262 (PMC5489152; doi:10.1371/journal.pone.0179262)

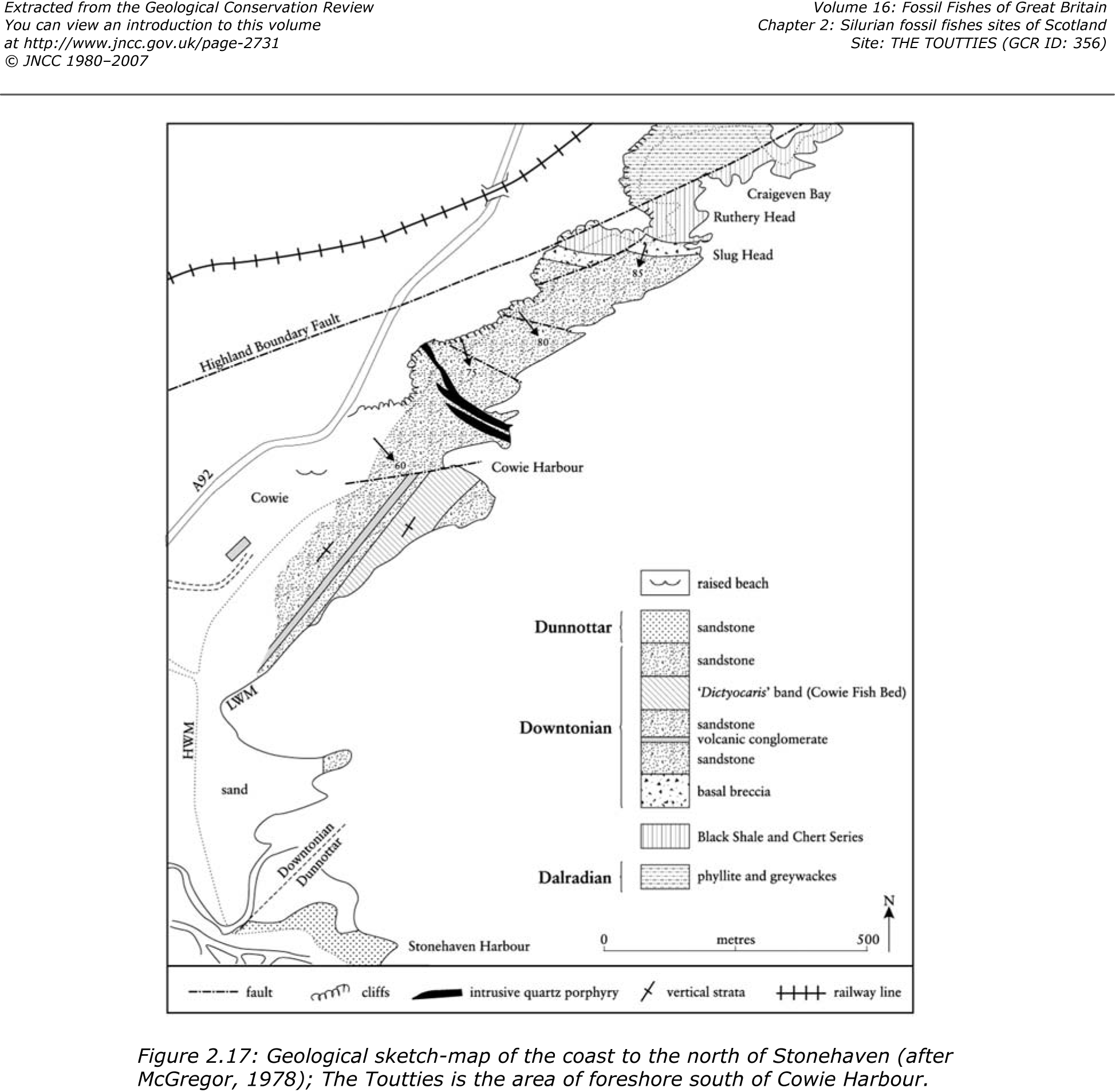

Supplement: S1 Fig — (TIF) [file pone.0179262.s002.tif]
